# Recurrent Wheezing in Pre-school Age: Not Only Airway Reactivity!

**DOI:** 10.3389/fped.2020.00101

**Published:** 2020-03-17

**Authors:** Marco Roversi, Federica Porcaro, Paola Francalanci, Adriano Carotti, Renato Cutrera

**Affiliations:** ^1^Academic Department, University of Rome Tor Vergata, Rome, Italy; ^2^Paediatric Pulmonology and Respiratory Intermediate Care Unit, Sleep and Long-Term Ventilation Unit, Academic Department of Paediatrics, Research Institute, Bambino Gesù Children's Hospital, Rome, Italy; ^3^Department of Pathology, Research Institute, Bambino Gesù Children's Hospital, Rome, Italy; ^4^Unit of Pediatric Cardiac Surgery, Research Institute, Bambino Gesù Children's Hospital, Rome, Italy

**Keywords:** pediatrics, wheezing, asthmatic bronchitis, bronchogenic cyst, airways abnormalities

## Abstract

**Background:** About a fifth of all mediastinal masses are primary cysts arising in the absence of other underlying pathology. Bronchogenic cysts, although rare, are the most frequent type responsible for lower airways compression as they often develop in the peripheral branches of the tracheobronchial tree.

**Case presentation:** We report the case of a 6-months-old child admitted for acute respiratory distress and wheezing not responsive to asthma treatment. Digestive and airway endoscopy proved a mild and a marked reduction of the esophageal and tracheal lumen, respectively. The nocturnal polygraphy showed an underlying obstructive disorder and the chest CT scan confirmed the presence of a wide mediastinal cyst compressing the trachea. The mass, later identified as a bronchogenic cyst, was surgically removed with complete resolution of the patient's respiratory symptoms.

**Discussion:** Our case shows that differential diagnosis of wheezing in pre-school aged children should encompass causes others than airway reactivity, thus prompting further evaluation and management.

## Background

**Figure 1:**
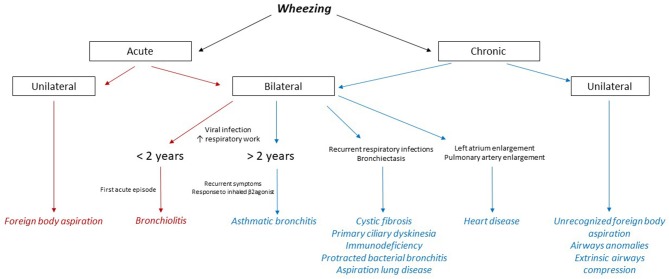
Flow chart on differential diagnosis of wheezing in children. The main causes of acute wheezing are highlighted in red, whereas the conditions underlying a chronic wheezing are colored in blue.

Despite being one of the most common finding in infants and children, wheezing never ceased to be an alarming symptom for both the parents and the physician. It consists of a continuous sound heard during normal expiration or inspiration when airways obstruction is severe (1). A wheezing sound is usually caused by turbulent airflow passing through a narrowed medium-sized airway. Particularly under pre-school age (<6 years), a heterogeneous group of diseases, ranging from a self-limited viral process to a life-threatening disease, can be responsible for this symptom (2, 3). Diagnosis and treatment of young children with wheezing can thus be challenging and assessment of any kind of wheezing should always include a careful examination and detailed medical history, comprising the time of onset and the concurrent clinical manifestations (Figure 1). A chronic wheezing unresponsive to any treatment should prompt further evaluation with advanced imaging as to exclude congenital anomalies of the tracheobronchial tree, comprising vascular rings and slings (4), or a mediastinal mass (Table 1). We discuss the case of an infant with persistent wheezing and acute respiratory failure due to a large mediastinal mass compressing the lower airways.

**Table 1:** Causes of recurrent/chronic wheezing in children.

Tracheo-bronchomalacia^*^
Vascular compression/rings^*^
Tracheal stenosis/web^*^
Extrinsic compression of trachea-bronchial tree (cyst or tumor, lymphadenopathy, cardiomegaly)
Asthma
Gastroesophageal reflux, aspiration
Not recognized foreign body
Bronchopulmonary dysplasia
Cystic fibrosis
Primary ciliary dyskinesia
Immunodeficiency
Bronchiolitis obliterans

* *These alterations tend to be present in pre-school aged children*.

## Case Presentation

**Figure 2:**
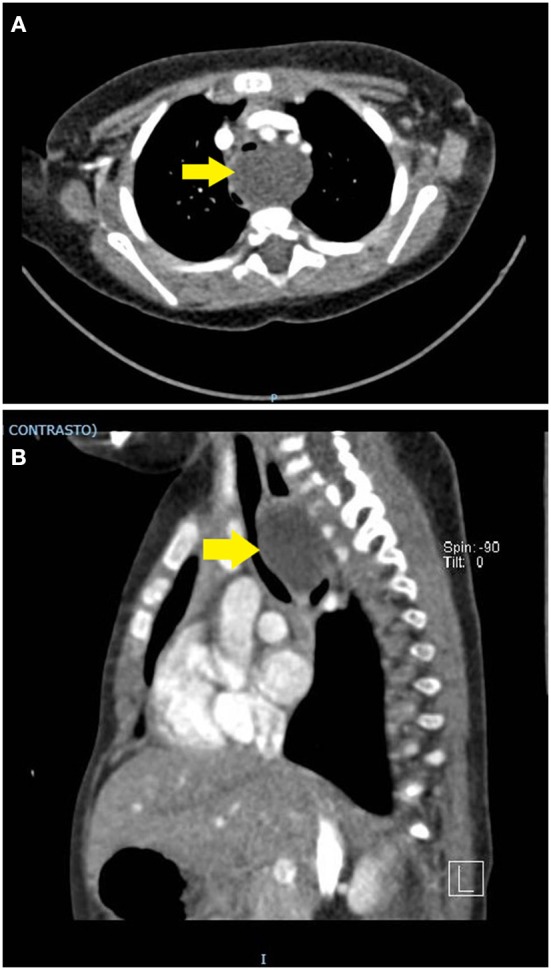
**(A,B)** Contrast-enhanced chest CT scan. A thin-walled round mass (antero-posterior to lateral diameter, size 30 × 25 × 40 mm) can be seen at the level of the first five dorsal vertebral somas (from D1 to D5). The mass (yellow arrow) occupies the upper and middle mediastinum, displacing the esophagus posteriorly and the trachea anteriorly. In dynamic acquisitions a marked reduction in the size of the trachea to the middle third is documented. The epiaortic vessels appear to be slightly displaced anteriorly without reduction in size.

A 6-months-old child was admitted at our hospital for acute respiratory failure. Her family history was positive for atopy. She was born at term from a vaginal birth and an uncomplicated pregnancy. Weight at birth was 2,790 g and respiratory distress in the immediate perinatal period was not reported. Recurrent asthmatic bronchitis not responsive to short course of inhaler bronchodilator and corticosteroids and growth retardation without clear symptoms of dysphagia (inability to swallow, regurgitation, gagging, cough during feeding) had occurred in the previous months. The parents reported a progressive worsening of the respiratory load. Physical examination showed dyspnoea, polypnea, prolonged expiration and wheezing worsening while eating. Chest X-ray revealed a normal tracheal air column and main stem bronchi with diffuse thickening of the bronchial walls, as for a non-specific inflammation of the bronchi and their surroundings. Blood tests and microbiologic analysis on respiratory secretions were negative. The echocardiography was limited by a poor acoustic window (due to significant air trapping) and was inconclusive for vascular rings. Based on the history of recurrent symptoms partially responsive to inhaler short term β2 agonists, the patient underwent airway and digestive endoscopy, which revealed a severe tracheomalacia at the T2-T3 level. Antero-posterior compression with a 1:1 ratio between the cartilage rings and the pars membranacea was observed. Anteriorly, the trachea appeared to be compressed by a pulsating mass. No abnormal communications between the airways and the digestive tract were found. Given the airway compression, a nocturnal polygraphy with overnight oximetry was carried out and proved the underlying obstructive disorder. In order to define the extrinsic compression and quantify the tracheal collapse, a dynamic chest CT scan with contrast enhancement was carried out and revealed a 4.0 cm wide mediastinal mass closely adherent to the anterior profiles of the first five thoracic vertebra, both compressing and dislocating the trachea and esophagus to the front and to the right, respectively (Figures 2A,B). Integration with ultrasound imaging directed at the jugulum confirmed the presence of a thin walled anechoic cyst. The patient underwent median sternotomy followed by opening of the pericardium and lateralization of the great vessels; total thymectomy was also made necessary to access the mass. On lowering the right pulmonary artery, the voluminous mass was appreciated, tightly adherent to the pars membranacea of the trachea and easily detachable from the esophagus. The cyst, filled with clear liquid and not communicating with the foregut, was therefore punched to reduce its size and facilitate dissection. Histopathological analysis later identified a cystic formation covered by respiratory epithelium and without smooth muscle in the walls, namely a bronchogenic cyst. The postoperative endoscopy revealed complete resolution of the tracheal compression at the T2-T3 level. Unfortunately, the patient had a severe respiratory distress with low oxygen saturation, requiring non-invasive ventilation in the intensive care unit (ICU) on the third postoperative day. Both wet and dry crackles could be heard at the right lower lobe. The chest X-ray confirmed the presence of a shaded consolidation at the right lower lobe, consistent with the expected postoperative dysventilation. An urgent endoscopy was required and proved the absence of any fistulous communication between airways and esophagus. Nevertheless, a paralysis of the left vocal cord was detected. Molecular analysis of the sputum later identified an infection by Rhinovirus. The patient respiratory function and imaging gradually improved until she was weaned from ventilatory support. She was discharged 3 weeks after surgery, with only residual expiratory sounds and dysphonia due to the left vocal cord paralysis. One month later, the patient's wheezing completely resolved, and the dysphonia also improved. We obtained informed written consent from the patient's parent authorizing publication of the clinical case and radiologic images. Her anonymity has been preserved.

## Discussion

About 20% of mediastinal masses are primary cysts arising in the absence of other underlying pathology (5). Unless they are of unspecified nature, primary cysts can originate from thymus, pericardium, digestive tract and airway system, with the latter being the most frequent type (6). Bronchogenic cysts arise from the precursor of the tracheobronchial tree, the respiratory diverticulum, as proven by their usual location proximal to the trachea or bronchi, either within the lung parenchyma or the mediastinum. In infants, they account for 10% of mediastinal masses and are more common in males (7). The cysts are usually identified by contrast-enhanced chest CT scan as well-defined homogeneous masses adjacent to the lower airways. Two thirds of bronchogenic cysts are asymptomatic, but surgical excision is always necessary as to confirm the diagnosis and rule out malignancies (6). Cyst resection is mandatory in children, even when asymptomatic, as they have a high risk of developing acute respiratory failure later in life, due to compression either of the trachea or bronchi (5). Furthermore, some cases show that half of the patients with bronchogenic cysts may develop complications, such as compression of adjacent structures, infection through a bronchial communication, and rupture into the trachea, the pericardial cavity, or the pleural cavity (8). Other complications of bronchogenic cysts are pneumothorax and pleuritis (9). In our case a common viral infection complicated a difficult postoperative course, given the age of the patient and the size of the cyst that had been removed. The kind of surgery that had been performed also had an intrinsic risk of causing iatrogenic tracheoesophageal fistula (10), thus making frequent endoscopic controls mandatory.

Despite the usual location of bronchogenic cysts within the mediastinum, the given one was well above the carina, compressing both the trachea and esophagus and leading to wheezing without feeding problems. On the basis of cyst's dimension, the symptoms were expected to appear earlier in life, but in our case no respiratory distress was noted at birth or immediately after. Treatment of mediastinal masses, such as the cyst we encountered, is hazardous and should always be managed by a multidisciplinary team. Along with bronchogenic cysts, there are a variety of causes of extrinsic compression of the trachea that may cause wheezing and mimic an asthmatic bronchitis in children, ranging from the more common mediastinal neoplasms to the less frequent vascular rings (11). Therefore, other causes than pre-school asthma have to be taken into account in younger children with wheezing poorly responsive to medical treatment. That is, not all pre-school wheezing means airway reactivity!

## Ethics Statement

We obtained informed written consent from the patient's parent authorizing publication of clinical case and radiologic imagings.

## Author Contributions

MR made data analysis, wrote the paper and approved the final manuscript. FP contributed to the writing of the paper, revised and approved the final manuscript. PF analyzed the bioptical specimen and made the final diagnosis, revised the paper and approved the final manuscript. AC performed the surgical removal of the cyst, revised the paper and approved the final manuscript. RC contributed to design, revised and approved the final manuscript.

### Conflict of Interest

The authors declare that the research was conducted in the absence of any commercial or financial relationships that could be construed as a potential conflict of interest.
